# The immune duality of osteopontin and its therapeutic implications for kidney transplantation

**DOI:** 10.3389/fimmu.2025.1520777

**Published:** 2025-02-28

**Authors:** Junto Leung, Lei Qu, Qifa Ye, Zibiao Zhong

**Affiliations:** ^1^ Zhongnan Hospital of Wuhan University, Institute of Hepatobiliary Diseases of Wuhan University, Transplant Center of Wuhan University, National Quality Control Center for Donated Organ Procurement, Hubei Key Laboratory of Medical Technology on Transplantation, Hubei Provincial Clinical Research Center for Natural Polymer Biological Liver, Wuhan, Hubei, China; ^2^ The 3rd Xiangya Hospital of Central South University, NHC Key Laboratory of Translational Research on Transplantation Medicine, Changsha, China

**Keywords:** osteopontin, immunology, kidney transplantation, SPP1, biomarker

## Abstract

Osteopontin (OPN) is a multifunctional glycoprotein with various structural domains that enable it to perform diverse functions in both physiological and pathological states. This review comprehensively examines OPN from multiple perspectives, including its protein structure, interactions with receptors, interactions with immune cells, and roles in kidney diseases and transplantation. This review explores the immunological duality of OPN and its significance and value as a biomarker and therapeutic target in kidney transplantation. In cancer, OPN typically promotes tumor evasion by suppressing the immune system. Conversely, in immune-related kidney diseases, particularly kidney transplantation, OPN activates the immune system by enhancing the migration and activation of immune cells, thereby exacerbating kidney damage. This immunological duality may stem from different OPN splice variants and the exposure, after cleavage, of different structural domains, which play distinct biological roles in cellular interactions. Additionally, OPN has a significant biological impact posttransplantation and on chronic kidney disease and, highlighting its importance as a biomarker and potential therapeutic target. Future research should further explore the specific mechanisms of OPN in kidney transplantation to improve treatment strategies and enhance patient quality of life.

## Highlights

- OPN exhibits immune duality, contributing to the development of an immunosuppressive microenvironment in tumors and, conversely, promoting tissue inflammatory responses in immune-related injuries.
- The immune duality of OPN may be attributed to its splice variants, with OPN-4/5 showing significant structural differences from OPNa/b/c, and thrombin cleavage further accentuates these disparities.
- Visualization analysis revealed that the RGD domain of CL-OPNa/b/c can bind tightly to integrins, whereas that of FL-OPNa/b/c cannot.
- OPN mediates immunocyte activities and polarization that facilitate acute and chronic rejection responses, as well as long-term fibrosis
- OPN has the potential to serve as a noninvasive biomarker for kidney transplant rejection, as it can be released into the urine through exosome-mediated mechanisms.

## 1 Introduction

Kidney transplantation is the most effective treatment for end-stage renal disease and significantly improves the quality of life and survival rate of patients (1). Despite continuous advancements in immunosuppressive agents and immunoinduction therapies, the incidence of both acute and chronic rejection remains high posttransplantation, with chronic rejection being the primary cause of graft failure (2–4). Several factors contribute to the occurrence and refractoriness of rejection, including ischemia−reperfusion injury (IRI), human leukocyte antigen (HLA) mismatches, and chronic activation of adaptive immune responses, all of which ultimately lead to graft dysfunction (5–7). Therefore, treating or preventing rejection remains one of the major challenges in the field of transplantation.

To treat rejection following kidney transplantation, a substantial amount of basic and clinical research has been conducted in the medical field. Increasingly, research has focused on proteins with immunoregulatory functions to address rejection (8–10). Osteopontin (OPN) has garnered increasing attention due to its diverse physiological functions in various tissues. Recent studies have indicated that OPN may serve as a marker for rejection after kidney transplantation and could be a therapeutic target for treating posttransplant rejection (11–13).

OPN is a member of the small integrin-binding ligand N-linked glycoprotein (SIBLING) family (14–17). Traditionally, the physiological function of OPN is to promote bone development during skeletal bone formation. However, it can also act as a chemokine, mediating the chemotaxis and activation of immune cells, thereby participating in immune regulatory responses (18). The role of OPN in the immune system exhibits significant duality: it can contribute to the formation an immunosuppressive microenvironment in the context of tumors while also exerting proinflammatory effects in other disease contexts (19–21).

The immunological duality of OPN complicates its specific mechanisms of action under different physiological and pathological conditions. Therefore, a thorough understanding of the physiological and pathological functions of OPN is crucial for the prevention and treatment of acute and chronic rejection following kidney transplantation. This paper reviews the structural characteristics and physiological functions of OPN, as well as the specific role of OPN in kidney transplantation. Additionally, we explore the role of OPN in the immune system, the mechanisms of action of OPN in posttransplant rejection, and the clinical significance of OPN.

## 2 Physiological basis of OPN

### 2.1 Gene mapping and subtypes of OPN

OPN, a multifunctional glycoprotein encoded by the secreted phosphoprotein 1 (SPP1) gene, is a member of the SIBLING family (14–17, 22). OPN can be synthesized by cells from various systems, including the urinary, immune, and skeletal systems. These cells include osteoclasts, osteoblasts, epithelial cells, endothelial cells, renal tubular epithelial cells, and various immune cells, such as T cells, NK cells, macrophages, and Kupffer cells (20, 23–26). Due to its diverse physiological functions in the immune system, such as the early activation of T lymphocytes, OPN was initially referred to as early T-lymphocyte activation 1 (ETA1) (16, 17).

Understanding the genetic background of OPN further elucidates its multifunctional roles. In 1989, M.C. Kiefer and colleagues identified the DNA sequence of human OPN, which includes a 67-base 5’ untranslated region, a 415-base 3’ untranslated region, and a 942-base coding region encoding 314 amino acids (27). Although the untranslated regions do not encode proteins, they play a crucial role in regulating gene expression. In 1990, M.F. Young and colleagues used Northern blot analysis to locate the OPN gene on the q22 region of human chromosome 4, and Southern blot analysis confirmed that this gene is a single-copy gene with a length ranging between 5.4 and 8.2 kb (28, 29). The determination of DNA localization provided important genetic background information for studying the function and regulatory mechanisms of OPN. In 1992, K. Kohri and colleagues identified seven exon fragments of the human OPN gene (30). According to databases such as NCBI and ENSEMBL, there is an additional DNA sequence within the intron fragments of OPN that can potentially be translated as an extra exon, although related research is limited.

In addition to the known exons, the regulation of the OPN gene involves various mechanisms, including regulatory elements in the promoter region. These elements respond to a variety of intracellular signals, thereby regulating the expression of OPN (31–33). The promoter region contains multiple transcription factor-binding sites that can be activated by various cellular signals, thus modulating the transcriptional activity of the OPN gene (31, 34, 35).

The complex regulatory network of the OPN gene ensures precise expression regulation across different cell types and physiological conditions. For example, the differential expression patterns of OPN in osteocytes, immune cells, and certain tumor cells indicate its complex role in maintaining bone health, regulating immune responses, and participating in tumor development (15, 31, 36).

There are three common subtypes of the OPN protein: OPNa, OPNb, and OPNc (27). Among these subtypes, OPNa is the most prevalent; although it is not the longest OPN transcript sequence, it can be translated into the longest OPN amino acid sequence (27, 37, 38).

Research on OPNb and OPNc is less extensive than that on OPNa. OPNb lacks exons 4 and 6 and can be translated into a protein consisting of 300 amino acids, including a signal peptide and a mature peptide segment. In contrast, OPNc lacks exon 4 and exon 5 and can be translated into a protein with 287 amino acids (39, 40). These splice variants may be expressed in specific cellular or pathological states. Compared to OPNa, OPNb and OPNc may have different roles in cell signaling and intercellular communication, thereby exhibiting distinct functions in specific disease contexts (39, 41). For example, OPNc, in contrast to OPNa and OPNb, is more effective at promoting macrophage migration (42).

In addition to these three common subtypes, there are two less studied subtypes of OPN: OPN-4 and OPN-5 (43, 44). OPN-4 lacks exons 4, 5, and 6 and can be translated into a protein consisting of 273 amino acids, including a signal peptide and a mature peptide segment. According to records in the NCBI database, OPN-5 mRNA is the longest splice variant of OPN, with 1750 base pairs. In addition to the seven common exon sequences, it includes an additional exon of 189 base pairs. However, the CDS of OPN-5 contains only 672 base pairs, translating into the shortest protein subtype of OPN, with only 224 amino acids.

Although current knowledge of OPN-4 and OPN-5 splice variants is relatively limited, lacking both depth and breadth of research, it can be inferred from the extensive biological activities exhibited by other OPN family members that OPN-4 and OPN-5 may also be involved in regulating biological processes such as cell adhesion, migration, immune response, and inflammation (44). Considering the high specificity and dynamic nature of OPN family member expression in specific cellular environments and pathological conditions, OPN-4 and OPN-5 may also be expressed only in certain cells or diseases, thereby affecting disease progression or immune cell infiltration (43, 45) For instance, Martins et al. (43) analyzed respiratory secretions from patients with severe influenza infection and found that the expression level of OPN-4 was significantly greater than that of other OPN splice variants. Unfortunately, research on the specific roles and expression patterns of OPN-4 and OPN-5 is limited.

**Figure 1:**
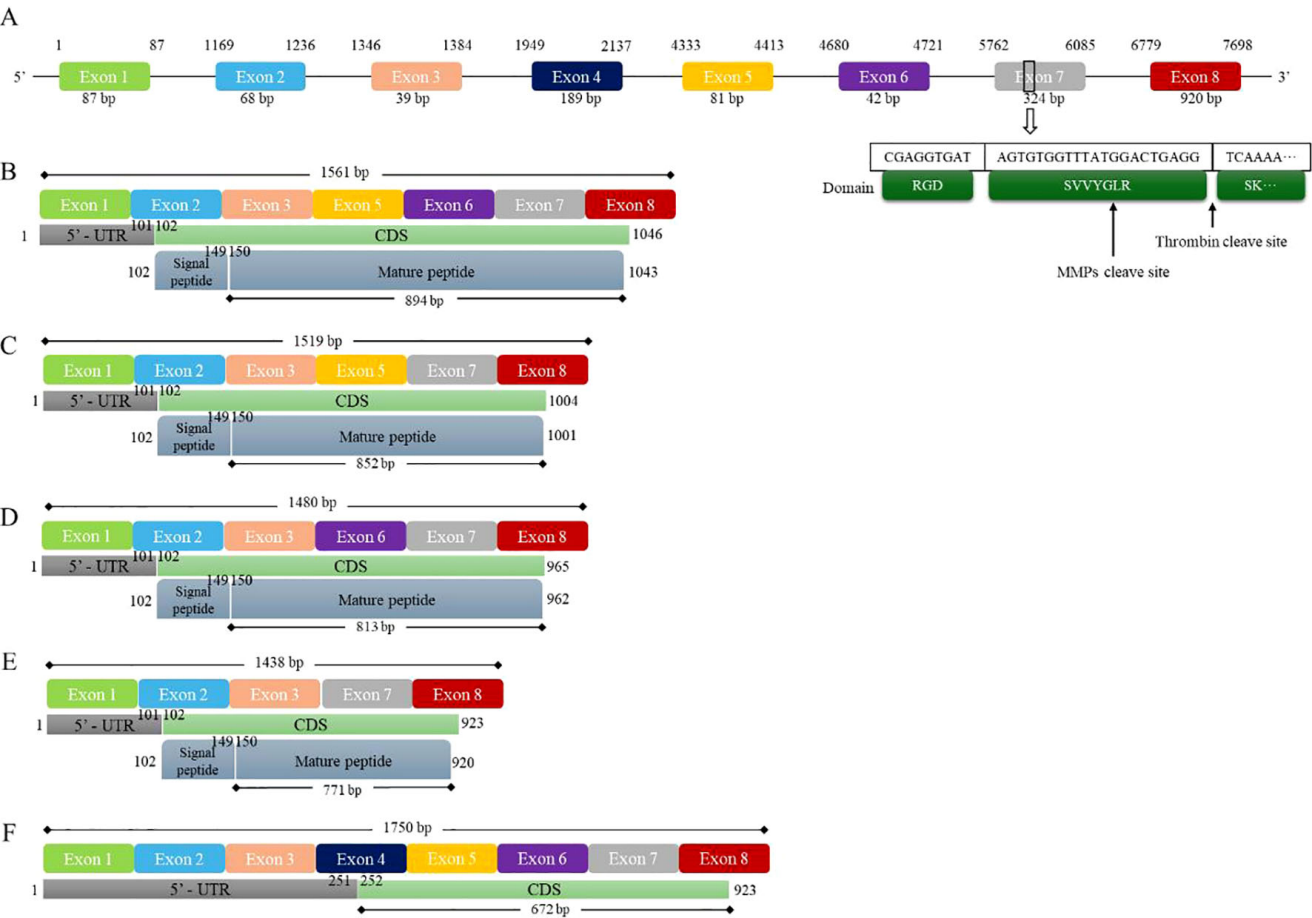
Five splice variants of human OPN and gene mapping. **Figure 1** shows the full DNA sequence and isoforms of human OPN. **(A)** The human OPN gene contains a total of 8 exons, with the 7th exon containing two important structural domains and a cleavage site. **(B)** The OPN-a isoform contains 7 exons, and 894 bp are translated into a 298 aa peptide. **(C)** The OPN-b isoform contains 6 exons, and 852 bp are translated into a 284 aa peptide. **(D)** The OPN-c isoform contains 6 exons, and 813 bp are translated into a 271 aa peptide. **(E)** The OPN-4 isoform contains 5 exons, and 771 bp are translated into a 257 aa peptide. **(F)** The OPN-5 isoform contains all 8 exons, and 672 bp are translated into a 224 aa peptide. CDS, coding sequence; MMPs, matrix metalloproteinases.

To investigate the relationship between transcription and translation among OPN subtypes, for this review, we downloaded the full-length OPN sequence and the transcript sequences of OPNa-c, OPN-4, and OPN-5 from the NCBI database. Using a custom-written Python script and the Biopython library, the sequence files were read and analyzed. The DNA sequences of the transcripts were aligned back to the original sequence to identify the position and length of each exon in the full-length OPN DNA sequence, as well as the specific locations of their CDS, signal peptide, and mature peptide (**Figure 1**) (46, 47). Additionally, in these studies, the three key domains of the OPN protein—the RGD domain, SVVYGLR domain, and thrombin cleavage site—were aligned and marked on the seventh exon of the OPN DNA sequence.

The various transcripts of OPN form the basis for the diverse biological functions of OPN proteins. These proteins provide flexibility and adaptability for OPN to regulate cellular behaviors and participate in complex pathological processes, allowing cells to precisely adjust OPN expression and function under different environmental conditions. Different OPN transcripts may perform distinct physiological functions. For example, the OPNa, OPNb, and OPNc subtypes are abnormally expressed in malignant tumors such as ovarian cancer, prostate cancer, breast cancer, and thyroid cancer (38, 40, 48, 49). Additionally, under certain inflammatory or stress conditions, specific splice variants may be preferentially expressed to adapt to environmental changes and exert particular biological effects (48, 50, 51). Although many studies have highlighted the functional differences among OPN transcripts, most current research on OPN has not delved deeply into the specific subtypes of OPN. Therefore, the precise expression and functional differences among OPN subtypes remain unclear.

### 2.2 Structure and domains of the OPN protein

The OPN protein is a phosphoglycoprotein with a molecular weight ranging from 44 kDa to 75 kDa (52, 53). In 1989, CW Prince predicted the secondary structure of the OPN protein through computational analysis, suggesting that it comprises eight α-helices and two β-sheets (54). The common form of OPN is monomeric, allowing it to freely circulate in the body, acting as a cytokine or binding to the extracellular matrix. However, under certain conditions, particularly pathological states, OPNa and OPNb can be cross-linked by transglutaminase to form multimers. This multimeric form may play a role in tissue repair, inflammatory responses, and tumor progression (55–57). Due to the absence of the necessary protein segment, OPNc cannot form polymeric complexes.

The main domains of the OPN protein include the RGD sequence, the SVVYGLR sequence, the thrombin cleavage site, and the calcium- and heparin-binding domains (12, 47, 52) Among these domains, the RGD sequence is one of the most important domains of the OPN protein. Arginine (R), glycine (G), and aspartic acid (D) are crucial sites for integrin binding (58). The RGD sequence plays a very important role in immunology, serving as a recognition or anchoring site for various cells. Under the action of cell membrane receptors, the RGD sequence links the extracellular matrix to the cytoskeleton, providing signals for cell anchoring, migration, or differentiation (59, 60).

The RGD domain can also interact with various integrins on the cell surface, such as those in the α4, α5, and α9 integrin families. These interactions can activate downstream signaling pathways, thereby playing a critical role in various biological processes, including cell adhesion, migration, invasion, and signal transduction (61, 62). An *in vitro* study revealed that macrophage-produced MMP3 can cleave the OPN protein, exposing the RGD domain, which induces lung fibroblast activation and promotes the progression of pulmonary fibrosis (63). Dai et al. (64) constructed an RGD sequence-containing nanogel *in vitro* and used it to treat osteoarthritis (OA) in mice. The results showed that the RGD nanogel could alleviate the progression of OA by blocking the binding of OPN to integrin β3 (64). Davaanyam et al. (65) constructed peptides containing partial sequences of the OPN protein *in vitro* and found that, compared to other peptides, the OPN peptide containing the RGD sequence most effectively regulated the immune function of mice and promoted macrophage polarization to the M2 phenotype. They also mutated the RGD sequence in this peptide, replacing RGD with RAA or RAD, and found that the RGD sequence plays a very important role in this peptide. In another study related to nerves and integrins, an *in vitro* constructed peptide containing the RGD sequence was used in mice and found to exert physiological effects equivalent to those of the OPN protein (66). Therefore, the RGD sequence is likely a key domain for the interaction between OPN and integrin molecules.

However, the RGD domain is usually embedded in the central part of the OPN protein. Therefore, the OPN protein needs to be cleaved by thrombin to expose the RGD domain and exert its physiological function of binding integrins (67). The thrombin cleavage site of the OPN protein is located between the R residue at the end of the RGDSVVYGLR sequence and the subsequent S residue (47). Peraramell et al. (67) used gene editing technology to knock in 153A into the mouse genome, constructing a gene-edited mouse resistant to thrombin cleavage. They found that these gene-edited mice exhibited characteristics similar to those of OPN knockout mice (67). This indicates that thrombin cleavage is an important step for the activation and function of OPN.

Thrombin cleavage not only exposes the RGD domain but also results in the formation of another important domain: SVVYGLR (47, 55, 68–70). The SVVYGLR domain consists of seven amino acids and primarily functions by binding to integrins α4β1, α4β7, and α9β1, thereby participating in various cellular behaviors, such as adhesion, migration, and invasion (71–73). The SVVYGLR domain is associated with several diseases, including skeletal diseases, inflammatory and autoimmune diseases, and cancer. Yamaguchi et al. (74) suggested that when the RGD sequence of the OPN protein is mutated, the SVVYGLR domain, which is exposed after thrombin cleavage, can substitute for the RGD domain in performing the physiological functions of a chemotactic factor. Additionally, SVVYGLR promotes angiogenesis and fibroblast differentiation (68). These physiological functions require thrombin cleavage.

Uchinaka et al. (70) used thrombin-cleaved OPN N-terminal fragments, C-terminal fragments, and N-terminal fragments lacking the SVVYGLR sequence in an *in vitro* study. They found that the OPN N-terminal fragment could significantly increase Smad signaling pathway activity and promote fibroblast migration (70). In contrast, the N-terminal fragment lacking the SVVYGLR sequence and the OPN C-terminal fragment did not have this function. In subsequent research, Ayako et al. (75) further demonstrated that the SVVYGLR sequence is a crucial domain for the functionality of the OPN N-terminal fragment.

In addition to these three most important domains, the OPN protein also contains several other protein domains, such as calcium-binding sites and heparin-binding sites, through which it exerts various physiological functions by binding to different molecules (76). For instance, when heparin binds to OPN, it can promote the unfolding of core elements in the OPN protein, which is also a crucial mechanism for the interaction between OPN and CD44 molecules (71, 77). *In vitro* studies have shown that heparin can increase the activity of the OPN protein (78). However, *in vivo*, this might also be related to the anticoagulant function of heparin (79). The calcium-binding site is an important site for OPN in regulating mineral deposition and bone remodeling, enabling OPN to play a bridging and regulatory role in bone tissue (80).

### 2.3 The main activation form of OPN: cleaved

OPN is a complex multifunctional protein that plays important roles in many key cellular processes, especially in various pathological conditions, such as inflammation, fibrosis, and tumors. The OPN protein can be cleaved by various enzymes, including thrombin and matrix metalloproteinases (MMPs), which produce different OPN fragments under the action of different enzymes. Each fragment may have unique biological activities and functions (63, 65, 71, 81). Full-length OPN is an uncut form that contains all its bioactive domains. Through interactions with integrins on the cell surface, it participates in cell adhesion, migration, and signal transduction. However, the two important domains of OPN are located in the central region of the protein. Compared to those of full-length OPN, cleaved OPN fragments, especially the OPN N-terminal fragment, exhibit more potent physiological functions (82, 83).

These cleaved fragments possess unique molecular structures and play crucial roles in the formation of the extracellular matrix, interactions between cells and the extracellular matrix, and cell signal transduction (47). In-depth research on the different cleavage fragments of OPN has improved our understanding of the multifunctionality and complexity of OPN in biological processes. The main cleavage sites of OPN are near the SVVYGLR fragment, with the thrombin cleavage site situated between the R residue at the end of the SVVYGLR fragment and the subsequent S residue and the MMP cleavage site located between the G residue and the L residue (47, 84, 85). The R residue at the end of the SVVYGLR domain is a key site for integrin α9 recognition of OPN. Therefore, when the OPN protein is cleaved by MMPs, it often cannot activate integrin α9 (85).

The various cleaved forms of OPN and its postcleavage fragments may be one of the reasons that OPN plays different roles in different disease contexts. Thrombin cleavage can activate the biological functions of OPN, with the resulting smaller fragments exhibiting different affinities for integrins, playing critical regulatory roles, particularly in inflammation, cell migration, and the tumor microenvironment (70). For example, in the tumor environment, the OPN N-terminal fragment produced by thrombin cleavage can inhibit the host local tumor immune response, thereby promoting tumor progression (67, 86). However, in the context of inflammation, the thrombin-cleaved OPN N-terminal fragment can induce dendritic cell (DC) adhesion (47). In cases of acute infection, the thrombin-cleaved OPN N-terminal fragment also upregulates proinflammatory cytokine expression and promotes the immune response (87). A clinical study on hypertension revealed that the OPN N-terminal fragment is associated with carotid atherosclerotic inflammation but is not related to the full-length OPN or the OPN C-terminal fragment (88). *In vitro* studies have shown that both full-length OPN and the OPN N-terminal fragment can induce NF-κB expression in macrophages and promote inflammation, whereas the OPN C-terminal fragment does not have this effect (89).

In addition to thrombin, the MMP family can cleave OPN (63). MMPs are a class of enzymes capable of degrading extracellular matrix proteins and can cleave OPN at different sites, producing various biologically active fragments. MMP-3 and MMP-7 have been shown to cleave OPN, generating multiple bioactive fragments (82, 90). Due to the different cleavage methods and sites of thrombin and MMPs, the exposed OPN amino acid residues differ, thereby allowing for different physiological functions (85).

The MMP cleavage site is within the SVVYGLR sequence, resulting in a cleaved N-terminal fragment lacking the L/R residues, which almost completely eliminates the function of the SVVYGLR domain in the OPN N-terminal fragment but retains the function of the RGD domain. This is the main reason why the N-terminal fragment of OPN cleaved by MMPs can continue to interact with integrins α5β3 and α5β1 (85). This finding also explains why the cleavage of the N-terminal fragment of OPN by MMPs can induce the activation of lung fibroblasts (63). In addition to MMPs, plasmin and cathepsin D can cleave OPN, increasing the accessibility of its key domains, enhancing receptor binding efficiency, and potentially conferring additional biological functions to OPN (82).

**Figure 2:**
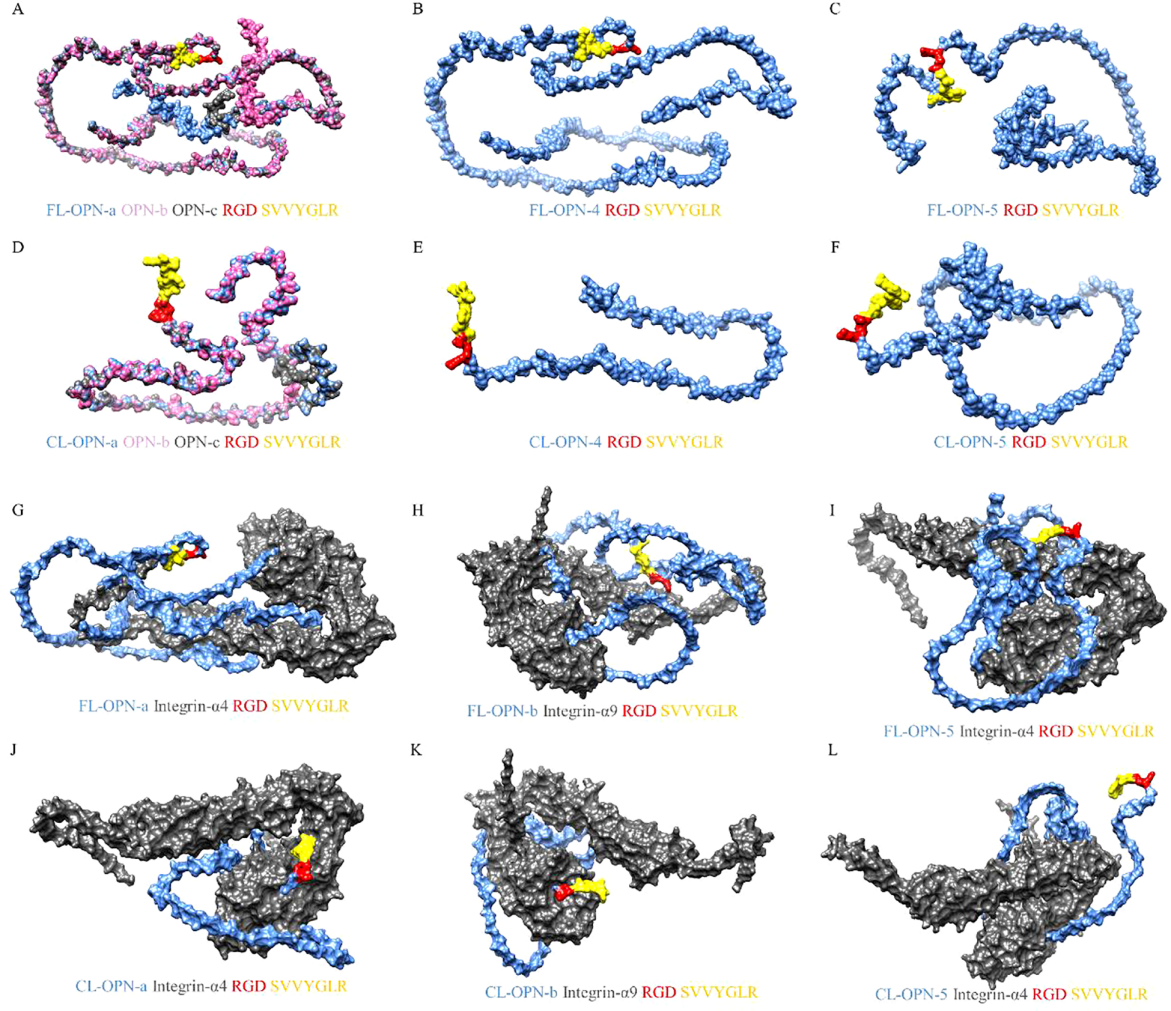
Visualization of OPN protein isoforms and interactions with integrins. **Figure 2** shows examples of OPN protein isoforms binding to two types of integrins. Visual analysis revealed that OPNa, OPNb, and OPNc exhibit similar protein structures, whereas OPN-4 and OPN-5 possess unique protein configurations. The distinctions among the isoforms become more pronounced upon thrombin cleavage. Following thrombin cleavage, the RGD domains of OPNa, OPNb, and OPNc can bind more tightly to integrins, whereas OPN4 and OPN5 demonstrate inadequate binding affinity to integrins, regardless of whether they have undergone thrombin cleavage. **(A)** The structures of the full-length OPN-a, OPN-b and OPN-c isoforms. OPN-a is displayed in blue, OPN-b is displayed in pink, and OPN-c is displayed in gray. **(B)** The structure of the full-length OPN-4 isoform. **(C)** The structure of the full-length OPN-5 isoform. **(D)** The structures of the thrombin-cleaved OPN-a, OPN-b and OPN-c isoforms. OPN-a is displayed in blue, OPN-b is displayed in pink, and OPN-c is displayed in gray. **(E)** The structure of the thrombin-cleaved OPN-4 isoform. **(F)** The structure of the thrombin-cleaved OPN-5 isoform. **(G)** The binding mode of the FL-OPN-a isoform and integrin-α4. **(H)** The binding mode of the FL-OPN-b isoform and integrin-α9. **(I)** The binding mode of the FL-OPN-5 isoform and integrin-α4. **(J)** The binding mode of the CL-OPN-a isoform and integrin-α4. **(K)** The binding mode of the CL-OPN-b isoform and integrin-α9. **(L)** The binding mode of the CL-OPN-5 isoform and integrin-α4. FL-OPN = full-length OPN; CL-OPN = cleaved OPN.

To gain a deeper understanding of the physiological functions of cleaved OPN, for this review, we used Biopython, SWISS-MODEL, and AlphaFold to predict the protein structures of the mature peptide segments of different OPN transcripts, the N-terminal fragments cleaved by thrombin, integrin α4, and integrin α9, as shown in **Figure 1** (46, 91, 92). Subsequently, GRAMM docking was used to analyze protein interactions between different OPN variants and integrins (93). Additionally, Chimera was used to visualize the results of the protein interaction analyses (**Figure 2**) (94). Visualization revealed that OPNa, OPNb, and OPNc have very similar protein structures, particularly the RGDSVVYGLR sequence. However, the protein structures of OPN4 and OPN5 show significant differences, especially in the N-terminal fragments cleaved by thrombin. These structural differences may be one of the reasons for the different physiological functions of different OPN subtypes.

Further visualization of the protein interaction analysis results showed that, compared to full-length OPN, thrombin-cleaved OPN exhibits close binding of its RGD and SVVYGLR domains with integrin α4 and integrin α9. This further confirmed that the RGD and SVVYGLR domains are critical for the physiological functions of OPN. However, for the OPN-5 subtype, whether in its full-length or cleaved form, its RGD sequence cannot bind to integrins. Subsequently, PDBe was used to further analyze the protein interaction results (**Table 1**) (95). The results showed that the binding capacity of OPNa, OPNb, and OPNc to integrin molecules generally increased after thrombin cleavage, whereas OPN-4 and OPN-5 did not exhibit this phenomenon. This indicates that compared to those of full-length OPN, the thrombin-cleaved forms of OPNa, OPNb, and OPNc are more likely to interact with integrins. Additionally, these findings suggest that the physiological functions of OPN-4 and OPN-5 may differ from those of the three classical OPN subtypes.

**Table 1:** Interactions between OPN isoforms and integrins (α4 and α9): binding energies (ΔG) and interface areas.

Integrin	OPN-a	OPN-b	OPN-c	OPN-4	OPN-5
△G (kcal/mol)					
α4					
FL-OPN	−11.5	−9.3	−1.5	−9.4	−18.6
CL-OPN	−20.7	−26.9	−18.4	−11.7	−7
α9					
FL-OPN	−23.7	−1.8	−1.2	−1.8	−8.5
CL-OPN	−18.0	−20.9	−11.6	−5.2	−5.5
Interface area (A^2^)					
α4					
FL-OPN	3413.2	2701.6	3087.3	2541.8	2696.0
CL-OPN	2795.2	3360.9	3019.1	2215.3	2319.1
α9					
FL-OPN	2527.9	2124.8	2196.5	2384.1	2300.0
CL-OPN	2996.4	3182.2	1912.5	2072.2	2241.1

ΔG, binding energy; CL-OPN, thrombin-cleaved OPN; FL-OPN, full-length OPN.

This table presents the binding energies and interface areas for five OPN isoforms, detailing interactions with integrins both in their full-length form and after thrombin cleavage. A smaller binding energy indicates tighter or more facile bond formation between the OPN isoforms and the integrins.

These molecular mechanisms help to better understand the roles of OPN in physiological and pathological processes, providing a foundation for developing therapeutic strategies. These studies not only reveal the multifunctionality of OPN but also offer new perspectives and possibilities for developing treatments for related diseases.

### 2.4 OPN receptors

OPN receptors include various cell surface adhesion molecules and integrins, among which integrins have multiple subtypes, such as α4, α5, and α9 integrins, all of which are target receptors for OPN. Integrins are widely expressed in various tissues and cells, including endothelial cells, macrophages, and certain tumor cells, such as melanoma and breast cancer cells. They are primarily responsible for interactions between cells and the extracellular matrix (96, 97). By binding to different integrins, OPN can exert multiple physiological functions, including (a) promoting the invasiveness of tumor cells and supporting tumor growth and metastasis; (b) facilitating fibroblast migration and the progression of tissue fibrosis; (c) enhancing immune cell infiltration and regulating the survival, proliferation, migration, and differentiation of immune cells; and (d) participating in tissue repair and inflammatory responses (98–102). These interactions and signaling networks highlight the crucial role of OPN in various biological and pathological contexts, making it an important target for the treatment of diseases related to abnormal cell migration and proliferation.

In the field of immunology, Simoes et al. (98) discovered that the SLAYGLR domain of murine OPN (corresponding to the SVVYGLR domain in human OPN) can bind to dendritic cells (DCs) via the α4β1 integrin, inducing DCs to produce interferon-β (IFN-β) through the PI3K/mTOR/IRF3 signaling pathway. In terms of angiogenesis, Osuka et al. (100) reported that the OPN N-terminal fragment forms part of the extracellular matrix and can promote the formation of new blood vessels by endothelial cells through the α9 integrin. In the context of tumors, OPN can activate the integrin α5 pathway, stimulating the FAK/AKT and ERK signaling pathways in tumor cells and thereby promoting the proliferation and drug resistance of non-small cell lung cancer (NSCLC) cells (102). The specific integrin receptor to which OPN binds is related to the cleavage method and domains of OPN; the RGD sequence can bind to α5 integrins, while the SVVYGLR sequence can bind to α4 and α9 integrins (85).

In addition to integrins, CD44 is also an important receptor for OPN, and OPN competes with α9 integrins for binding to CD44 (31, 71). CD44 is a glycoprotein expressed on various cell surfaces, mainly on macrophages, T lymphocytes, and B lymphocytes, and is closely related to cell adhesion, migration, and signal transduction processes (18, 103, 104). The binding of OPN to CD44 can significantly upregulate the expression of intracellular p-CaMKII, p-RyR, and p-PLN (105); it also promotes the migration and activation of immune cells such as T cells and macrophages, thereby enhancing the inflammatory response (31, 106). This process is crucial for the rapid response and effective defense mechanisms of the immune system. CD44 has multiple isoforms, and its ability to bind to OPN varies, with the V9-V10 isoform having a relatively stronger binding capacity to OPN (107). The absence of CD44v7 in macrophages can reduce OPN-mediated colonic inflammation (108). In summary, OPN regulates the migration and adhesion of various cell types through the CD44 receptor, which is particularly important in tumor progression and metastasis, tissue repair, the inflammatory response, and fibrosis.

## 3 Interaction between OPN and immune cells

### 3.1 Interaction between OPN and T/B lymphocytes

T lymphocytes play a crucial role in tumors, infections, and organ transplant rejection through various mechanisms, such as differentiation, direct cytotoxicity, and immune regulation (109). In recent years, an increasing number of studies have revealed the complex and important relationships and mechanisms between OPN and T lymphocytes. However, these studies also highlight the significant duality of OPN in regulating immune functions. For example, OPN can induce an immunosuppressive tumor microenvironment in the context of tumors, while it can induce an inflammatory and fibrotic microenvironment in the context of inflammation. This may be related to the different subtypes or cleavage methods of OPN that can activate different receptors on the surface of T lymphocytes, or it may be related to the localization of the functional activity of OPN (85, 110, 111). For instance, a study by Masashi et al. (110) revealed that intracellular OPN can reduce the number of myeloid cells, whereas extracellular secreted OPN can increase the number of lymphocytes.

OPN can primarily mediate T cell activation, differentiation, and downstream signaling pathway expression through integrins and CD44 receptors on the surface of T cells. By regulating T cell function and the local immune microenvironment, OPN influences disease progression and the course of the disease (31, 112). In certain types of diseases, such as multiple sclerosis, local tissues can produce large amounts of OPN to inhibit T cell activity, reducing the production of cytokines such as IL-2, TNF, and IFNγ by T cells and thereby creating an immunosuppressive microenvironment (113). Similarly, in some tumors, tumor cells with high OPN expression can inhibit T cell function through CD44. Treatment with anti-OPN or anti-CD44 antibodies can reverse the inhibition of T cell activity by tumor cells, thus inhibiting tumor growth (114). Therefore, OPN may function as an immune checkpoint in the immune system, regulating T cell activation (115).

OPN not only affects the overall activity of T lymphocytes but can also specifically regulate different T cell subpopulations. For example, regulatory T cells (Tregs) can be regulated by OPN through the CD44 signaling axis, affecting the number of Tregs (116). Changes in the CD44-OPN signaling axis may be one of the reasons for the changes in T lymphocyte subpopulations (116, 117). Zhao et al. (118) reported that treatment with OPN *in vitro* can significantly inhibit Treg differentiation and promote Th1 cell differentiation, exacerbating autoimmune myasthenia gravis. However, OPN can also bidirectionally regulate the Th1/Th2 balance. A clinical study on rheumatoid arthritis (RA) revealed that active RA patients had elevated serum OPN levels and increased absolute Th2 cell counts, while the absolute Th1 and Treg cell counts decreased (119). In a study on allergic airway inflammation, OPN levels were also found to be positively correlated with Th2 cell levels (120). The activation of Th1 cells also depends on the function of DCs, and the adhesion of DCs is regulated by OPN (47, 121). In DCs, the absence of OPN leads to the downregulated expression of IL-12 and other costimulatory molecules (121). This may be one of the mechanisms by which OPN mediates T cell differentiation through DCs. In addition to regulating the differentiation of T lymphocyte subpopulations, OPN can also activate the CD44v7 receptor on T lymphocytes to inhibit T lymphocyte apoptosis. This might be another important mechanism by which OPN regulates the immune microenvironment through the CD44 molecule (108).

In addition to CD44, OPN can mediate proinflammatory or anti-inflammatory phenotypes of immune cells through other receptors on the surface of T cells. Wei et al. (122) conducted a receptor−ligand pairing analysis through a large-scale clinical single-cell transcriptomic analysis and found that the OPN-PTGER4 ligand−receptor pair is associated with CD8+ T lymphocyte dysfunction, immunosuppressive states, and late-stage tumor development. Prostaglandin E receptor 4 (PTGER4) is a receptor for prostaglandins and is widely expressed in T cells and macrophages (123–125). PTGER4 can downregulate the expression of the IL-2Rγc chain, inhibiting the response of tumor-infiltrating lymphocytes (TILs) to IL-2 in tumor tissues; this leads to a defect in the assembly of the IL-2Rβ-IL-2Rγc dimer on the cell membrane, thereby inhibiting the response of CD8+ T cells to IL-2 (126).

However, OPN does not inhibit T lymphocytes in all diseases. For example, in intestinal immune responses, the absence of OPN leads to a reduction in the number of CD4+ T lymphocytes and CD8+ T lymphocytes (127). This finding suggests that in the context of inflammation, OPN may promote the infiltration of T lymphocytes into tissues. *In vitro* experiments have also shown that supplementation with OPN can promote the survival of T lymphocyte subpopulations (127). In studies on Parkinson’s disease, OPN was found to promote the migration of CD4+ T lymphocytes (128). In the development of metabolic-associated fatty liver disease, high expression of OPN also promotes T lymphocyte infiltration, while knocking out the OPN gene can inhibit the polarization of T lymphocytes to Th1 cells (129). In chronic tissue inflammation, an increase in OPN promotes T lymphocyte infiltration and enhances the expression of IL-17a and TNF-α by T lymphocytes (130). Shirakawa and Sano (131) suggested that in chronic inflammation caused by obesity, OPN secreted by T cells promotes a chronic inflammatory response. This may be because, in this inflammatory context, senescent T cells express large amounts of OPN, further promoting the inflammatory response (132). Immunization with OPN peptides or OPN monoclonal antibodies can inhibit this inflammatory response by blocking the interaction between T cells and cleaved OPN (133).

In organ transplantation, OPN can promote the activation, migration, and tissue infiltration of CD8+ T cells through synergistic action with the T cell receptor/CD3 signaling pathway (134). Conversely, a reduction in the number of CD8+ T lymphocytes can lead to the decreased expression of OPN in tissues (135). Studies on kidney transplantation have also shown that OPN can recruit and activate immune cells, particularly T cells and macrophages, through chemotactic effects, thereby exacerbating rejection or promoting immune infiltration (136–138). These studies indicate that OPN can bidirectionally regulate the inhibition and activation of T lymphocytes in tumor diseases and other conditions. The specific reasons may be related to the cleavage of OPN, its subtypes, or the disease context, but relevant research is still lacking.

Studies on the relationship between OPN and B cells are relatively limited, but existing research suggests that the influence of OPN on B cells may be mediated through T cells. Research indicates that OPN can promote B cell proliferation and antibody production by enhancing T cell proliferation, interferon production, and CD40 ligand expression (139). OPN also plays a role in various B cell-mediated diseases, particularly in the immune response following infection; it regulates B cell activation and differentiation, affecting antibody production, suggesting that it may play a significant role in regulating B cell-mediated immune memory and protective immune responses. However, B cells can also produce OPN and participate in anti-malarial infection responses (140). *In vitro* experiments have shown that OPN can downregulate the expression of the costimulatory molecules CD80 and CD86 on the surface of B cells, and that B cells treated with OPN produce significantly less IL-6 (141). Intracellular OPN can act as a brake on the TLR9 signaling pathway, maintaining the normal physiological functions of B lymphocytes (142).

### 3.2 Interaction between OPN and macrophages

The regulation of macrophages by OPN usually leads to changes in the immune microenvironment (143, 144). In tumor diseases, OPN+ macrophages exhibit protumor characteristics, and their infiltration into tissues increases as the tumor progresses (145–147). However, the increased infiltration of OPN+ macrophages can inhibit T cell function, thereby promoting tumor cell progression and contributing to the development of fibrosis in the microenvironment (148, 149). This may be related to OPN+ macrophages promoting the differentiation of T cells into Tregs through CD44 (117). Therefore, some scholars believe that OPN+ macrophages have immunosuppressive properties (150). However, further analysis of OPN+ macrophages is needed. In further studies on OPN+ macrophages, Li et al. (146) reported that OPN+CD209- macrophages can promote angiogenesis and that OPN+CD209+ macrophages exhibit phagocytic functions. This finding suggests that, in addition to OPN, other cytokines or surface receptors may play a role in tumor-associated macrophages.

Hong et al. (151) utilized single-cell sequencing technology and discovered that the number of OPN+ macrophages increases locally in tumors and is associated with poor prognosis in colon cancer patients. This may be because OPN+ macrophages highly express Nicotinamide phosphoribosyltransferase (NAMPT), which is an inducible gene that mediates macrophage activity and IFN response. Li et al. (152) suggested that the relationship between OPN+ macrophages and poor prognosis in tumor patients might also be mediated through the CD44 molecule, as the OPN-CD44 signaling axis significantly enhances cell communication between macrophages and epithelial cells (152). The disruption of OPN+ macrophages can improve the efficacy of tumor treatments (53, 153). Additionally, in tumors, OPN can exert antiapoptotic effects through the CD44 receptor (154).

OPN+ macrophages can also exhibit a profibrotic phenotype (155, 156). IL-6 and TGF-β increase the number of OPN+ macrophages and are also factors that induce the formation of an immunosuppressive microenvironment (114). This immunosuppressive microenvironment may be related to the local formation of fibrous structures by interleukins and TGF-β, which restricts the entry of T cells (129, 153). In addition to OPN produced by macrophages themselves, OPN produced by tumor endothelial cells locally promotes the conversion of macrophages to an immunosuppressive M2 phenotype (157). Interventions using drugs or genetic engineering can reverse macrophage-mediated immunosuppression and enhance T cell infiltration and activation (157). This may be related to the binding of tumor cell-expressed OPN to macrophage PTGER4, as OPN can mediate crosstalk between tumor cells and macrophages through PTGER4, leading to macrophage polarization to the M2 phenotype (124, 158). In some acute infectious diseases, OPN also exerts similar immunosuppressive effects on macrophages. Hansakon et al. (159) found in a mouse model of fungal lung infection that OPN can induce macrophage polarization to the M2 phenotype and that OPN knockout enhances the ability of mouse macrophages to clear fungi.

OPN+ macrophages also play a role in promoting tumor metastasis (160). The specific mechanism may be related to SLC2A1, which can inhibit T cell function by increasing the infiltration of OPN+ macrophages, thereby creating an immunosuppressive microenvironment in tumor tissue and promoting tumor immune evasion (161). A clinical study on skin tumors also revealed that local T cell exhaustion and OPN+ macrophage infiltration are characteristics of tumor recurrence (146).

These studies collectively indicate that in the tumor environment, OPN tends to mediate the formation of an immunosuppressive microenvironment, particularly involving OPN produced by tumor cells and macrophages with high OPN expression. However, this effect is not solely related to OPN; it may also involve the synergistic effect of CXCL9 and OPN (162). The mutually exclusive expression of CXCL9 and SPP1 in the TME not only determines TAM polarity but also shows strong correlations with immune cell characteristics, antitumor factors, and patient prognosis, significantly impacting outcomes. Therefore, it is generally believed that increased OPN expression in macrophages within tumor tissues indicates the formation of an immunosuppressive microenvironment, whereas reduced OPN expression in macrophages is a marker of a proinflammatory immune environment (163–166).

Studies suggesting that OPN+ macrophages have immunosuppressive functions are mostly tumor-related. However, not all OPN+ macrophages mediate the formation of an immunosuppressive microenvironment. In many other diseases, OPN can also mediate macrophage polarization toward the proinflammatory M1 phenotype (167, 168). For example, in chronic cardiovascular diseases, circulating OPN or OPN+ macrophages can interact with CD44/integrin on adipose progenitor cells, promoting macrophage polarization to the M1 phenotype and stimulating the development of atherosclerosis (169–171). A meta-analysis of patients undergoing hemodialysis also revealed that serum OPN levels are positively correlated with cardiovascular event mortality (172); this may be related to the promotion of atherosclerosis by OPN, although the authors did not specify the exact causes of death in the included population. In the context of skin inflammation and osteoarthritis, both secreted OPN and intracellular OPN can promote the inflammatory phenotype of macrophages (168, 173).

In ischemia-related diseases, OPN+ macrophages upregulate the MAPK signaling pathway and shift to a proinflammatory phenotype (174). Unlike in tumor diseases, the proinflammatory phenotype of macrophages in ischemia-related diseases can exacerbate tissue damage by inducing inflammation (175). Zhang et al. (176) also reported that after IR injury, macrophage polarization to the M2 phenotype can regulate the immune microenvironment, restore IL-10 secretion, and alleviate tissue damage. In such ischemic injuries, OPN can also interact with ischemia-activated platelets to promote macrophage expression of various cytokines (177). OPN can also induce monocyte expression of MCP-1, mediating the migration and infiltration of inflammatory cells (178). These findings indicate that in diseases unrelated to tumors, OPN or OPN+ macrophages usually polarize toward the proinflammatory M1 phenotype, exacerbating tissue damage by promoting immune responses.

However, some studies have shown that M2 macrophages can also play a role in ischemia-related diseases. Shirakawa et al. (179) demonstrated, using luciferase reporter gene transgenic mice, that after myocardial infarction, M2 macrophage infiltration increases at the infarction site and that almost all the OPN in the tissue is produced by M2 macrophages. These M2 cells can promote myocardial fibrosis and clear apoptotic cells to facilitate tissue repair. In NASH, OPN secreted by hepatocytes can mediate a macrophage-mediated inflammatory response through paracrine signaling, accelerating the progression of NASH (180).

**Figure 3:**
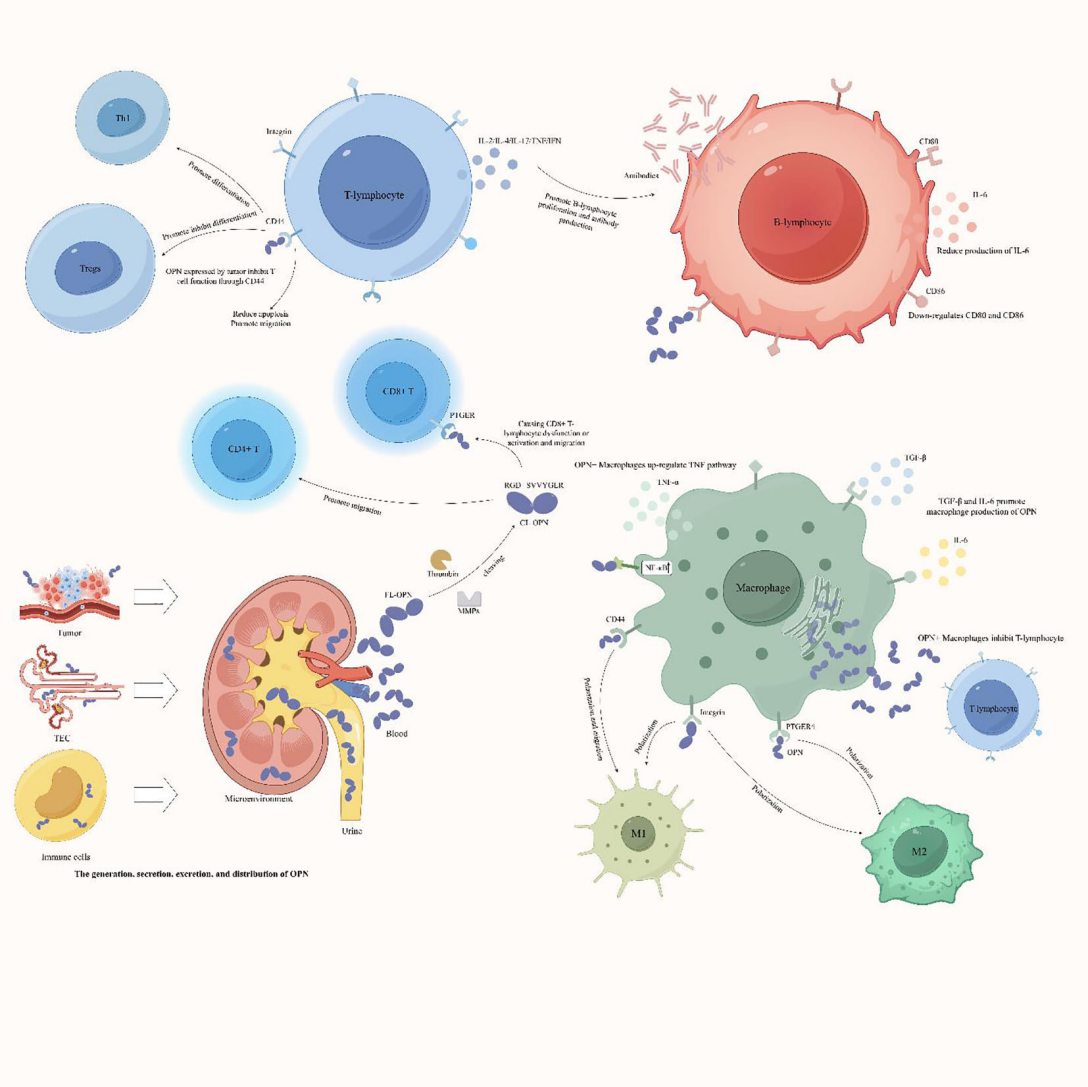
Complex network interactions between the OPN protein and immune cells. The OPN protein can be expressed by renal tubular epithelial cells, immune cells, and tumor cells. After the intact OPN protein is cleaved by thrombin or matrix metalloproteinases (MMPs), the RGD and SVVYGLR domains are revealed and bind to specific receptors on immune cells. This interaction regulates the chemotaxis, differentiation, or polarization of immune cells and mediates the expression of other proinflammatory factors by immune cells. Consequently, OPN plays a role in promoting the formation of an immunosuppressive microenvironment or proinflammatory immune responses. Th1, T helper 1 cell; Tregs, regulatory T cells; TEC, tubular epithelial cells; FL-OPN, full-length OPN; CL-OPN, cleaved OPN; PTGER, prostaglandin E receptor; MMPs, matrix metalloproteinases.

OPN+ macrophages can play different roles in various diseases, and secreted OPN can mediate macrophage polarization to either M1 or M2, thereby promoting or inhibiting the immune microenvironment. Most studies suggest that OPN+ macrophages polarize toward the M2 phenotype in tumor tissues, creating an immunosuppressive microenvironment that promotes tumor progression and escape. However, in other diseases, OPN+ macrophages polarize toward the M1 phenotype, inducing immune infiltration and promoting immune damage. The specific underlying mechanisms are not yet well understood. To better illustrate the interactions between OPN and immune cells, we used the FigDraw platform to create a mechanism diagram (**Figure 3**) depicting the various regulatory mechanisms of OPN on immune cells in different disease contexts. This illustration helps to better understand the dual role of OPN in immunology.

## 4 OPN and kidney diseases

### 4.1 OPN and kidney injuries

**Figure 4:**
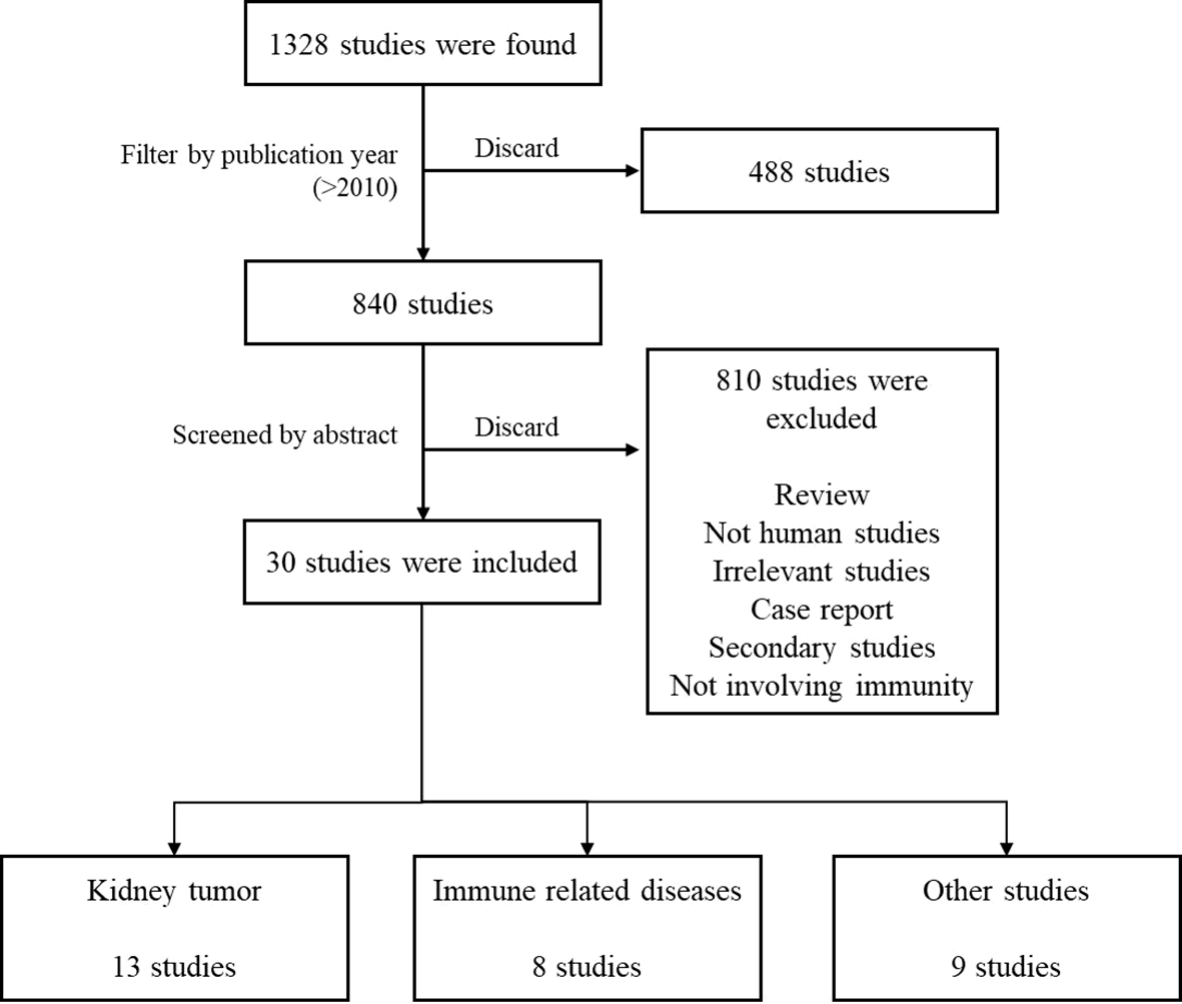
Flow chart of the literature search. **Figure 4** illustrates the literature search process, through which 30 relevant studies were selected from a total of 1,328 publications. Among these, 13 focused on kidney tumors, 8 addressed immune-related diseases, and 9 covered other renal diseases.

The relationship between the kidneys and the immune system is close, with immune dysfunction being an initial factor in many kidney diseases, such as IgA nephropathy and lupus nephritis. Even diabetic nephropathy ultimately leads to renal fibrosis due to immune infiltration. Therefore, understanding the functions and roles of OPN and immune cells in kidney diseases is crucial for understanding the mechanisms of OPN and immune cells after kidney transplantation. For this review, we used the search string “((kidney[Title/Abstract]) OR (renal[Title/Abstract])) AND ((SPP1[Title/Abstract]) OR (Osteopontin[Title/Abstract]))” to retrieve studies on OPN and human kidney diseases from the PubMed database published between January 1, 2010, and June 1, 2024. From the retrieved articles, 30 studies involving human samples were selected based on their abstracts (**Figure 4**). These studies were classified into categories: 13 studies on kidney tumors, 8 studies related to immune kidney diseases, and 9 studies on other kidney diseases. The key content from these studies was extracted to further explore the role of OPN in kidney diseases (**Table 2**).

**Table 2:** Clinical studies on OPN and kidney diseases (2010–2024).

Study identifier	Disease type	Promote or inhibit immune environment	Sampling	Source of OPN	Changes in immune cells	Role and mechanisms of OPN
Xu et al. (181)	Tumor	Inhibit	Tissue	Tumor tissue	Reduction in CD8+ T-lymphocytes/increase in Tregs	OPN promotes tumor immune evasion by inhibiting CD8+ T lymphocyte infiltration and enhancing Tregs cell infiltration.
Zhang et al. (182)	Tumor	Inhibit^*^	Tissue	Tumor cells	–	OPN binds to integrin receptors on the surface of target cells in renal cell carcinoma, activating signaling pathways such as JAK/STAT, which in turn facilitates disease progression.
Chen et al. (185)	ORG	Promote	Tissue	Increased expression of OPN in T-lymphocytes and B-lymphocytes	Decreased number of T lymphocytes and B lymphocytes	Although lymphocyte infiltration is reduced, the increased expression of OPN in lymphocytes suggests that OPN may promote inflammatory responses and disease progression in ORG.
Ye et al. (186)	FSGS	Promote	Tissue/urine	TECs, podocytes, and macrophages	Increased macrophage infiltration and M2 polarization	OPN is positively correlated with the accumulation and M2 polarization of macrophages.
Steinbrenner et al. (210)	CKD	–	Urine	–	–	Higher OPN levels are associated with a higher risk for adverse outcomes.
Kim et al. (266)	AKI	–	Blood	–	–	–
Armstrong et al. (183)	Tumor	Promote	Blood	–	Enhancement of T-cell differentiation toward Th17 cells	OPN promotes the differentiation of T cells into Th17 cells and induces IL-17 production.
Xu et al. (232)	AR	Promote^*^	Blood	–	–	OPN promotes endothelial cell proliferation.
Owens et al. (211)	CKD	–	Urine	–	–	OPN can serve as a predictive marker for the prognosis of CKD.
Almaani et al. (267)	LN	Promote^*^	Tissue	Kidney tissue	–	–
Wang et al. (35)	Tumor	–	Tissue	Tumor tissue	–	OPN enhances the migration and invasion of tumor cells by upregulating NF-κB expression.
Kaleta et al. (187)	IgAN	Promote^*^	Urine	Kidney tissue^*^	–	Due to genetic polymorphisms of OPN, urinary excretion of the rs1126616 and rs9138 subtypes of OPN is significantly elevated in patients with IgAN.
Spinelli et al. (188)	LN	Promote^*^	Blood/urine	Kidney tissue^*^	–	Plasma levels of OPN are significantly higher in patients with active LN (lupus nephritis) and in those with LN remission than in healthy normal controls.
Ibrahim et al. (191)	AKI	Promote^*^	Urine	–	–	Drug-induced kidney injury can increase the level of OPN in urine.
Choueiri et al. (184)	Tumor	Inhibit^*^	Blood	–	–	Lower levels of OPN in the blood can enhance the therapeutic efficacy for RCC patients.
Sobotke et al. (268)	Tumor	Inhibit^*^	Blood	–	–	The serum levels of OPN in RCC (renal cell carcinoma) patients are significantly higher than those in controls, and they correlate with the size and stage of the tumor. High levels of OPN are associated with poorer overall survival (OS) rates and cancer-specific survival (CSS).
Berti et al. (269)	AAV	Promote^*^	Blood	–	–	High levels of OPN may be associated with disease activity and prognosis in MPA patients.
Zurita et al. (270)	Tumor	Inhibit^*^	Blood	–	–	High levels of OPN are correlated with poorer overall survival.
Trojanowicz et al. (271)	Hemodialysis	Promote^*^	Blood	–	–	–
Taguchi et al. (213)	Urolithiasis	Promote	Tissue	RPs, TECs, and interstitial cells	Increased infiltration of macrophages, plasma cells, and neutrophils	High expression of OPN can promote calcium salt deposition and crystallization, thereby facilitating the formation and progression of RP.
Brooks et al. (272)	Urolithiasis	Promote^*^	Urine	–	Increased MIP-1α in urine (secreted by macrophages)	OPN can protect the kidneys from oxalate crystal-induced injury.
Ma et al. (273)	LN	Promote	Tissue	Kidney tissue	Increased infiltration of T-lymphocytes and macrophages	OPN facilitates macrophage infiltration within the kidney and induces injury to podocytes, leading to proteinuria.
Rabjerg et al. (274)	Tumor	Inhibit^*^	Tissue	Kidney tissue	–	High expression of OPN is associated with poorer survival outcomes.
Al-Malki (209)	DN	Promote^*^	Urine	–	–	There is a positive correlation between the number of podocytes in urine and the level of OPN.
Okumura et al. (275)	Urolithiasis	–	Stone	–	Kidney stones contain a variety of proteins enriched with neutrophils	OPN protein is present in most kidney stones.
Righi et al. (276)	Tumor	Inhibit^*^	Tissue	Tumor tissue and TECs	Increased infiltration of macrophages in tumor tissue	OPN may indirectly contribute to the mechanisms driving RCC progression and impact patient survival by influencing aspects such as the invasiveness, proliferation, and treatment resistance of tumors.
Jin et al. (231)	AR	Promote^*^	Blood	–	–	During acute rejection episodes, the levels of OPN in the blood increase, and they decrease following anti-rejection therapy.
Sim et al. (277)	Tumor	Inhibit^*^	Blood	–	–	Higher concentrations of OPN in the blood are associated with poorer survival outcomes in patients with RCC.
Papworth et al. (278)	Tumor	Inhibit^*^	Blood	–	–	Blood OPN levels are an independent prognostic factor for RCC-specific survival.
Zurita et al. (279)	Tumor	–	Blood	–	–	Patients with higher concentrations of OPN in the blood tend to derive greater benefits from targeted treatments.

AAV, antineutrophil cytoplasmic antibody-associated vasculitis; AKI, acute kidney injury; AR, acute rejection; DN, diabetic nephropathy; FSGS, focal segmental glomerulosclerosis; IgAN, IgA nephropathy; LN, lupus nephritis; OPN, osteopontin; ORG, obesity-related glomerulopathy; OS, overall survival; RP, Randall plaques; TEC, tubular epithelial cell. ^*^Not explicitly stated in the original study but inferred from cytokines and medical changes.

These clinical studies revealed that OPN generally promotes tumor progression or metastasis in kidney tumors. Specifically, OPN can enhance Treg infiltration and M2 macrophage polarization, significantly promoting an immunosuppressive microenvironment (181–184). However, in immune-related and other kidney diseases, OPN can exacerbate disease progression by activating and sustaining inflammatory responses (185–188). This immunological duality may be related to various factors. For example, Silver and Popovics (26) suggested that in prostate tumors, OPN expressed by prostate tumor cells is the main reason for promoting tumor progression and invasion and that in benign prostate diseases, OPN secreted by macrophages is the main reason for promoting inflammation and fibrosis. However, these clinical studies did not provide detailed distinctions regarding the source, subtype, or cleavage forms of OPN, which remains an important issue for further research.

In the kidneys, tubular epithelial cells themselves can produce OPN and participate in regulating kidney repair and regeneration processes (189). For example, in acute kidney injury (AKI), the expression of OPN is rapidly upregulated as a natural response to kidney damage (190, 191). This may be related to the regulation of OPN by ROS generated by AKI through the NF-KB/RUNX2 or other signaling pathways (192–194).

The progression of chronic kidney disease is also accompanied by mineral bone disease (195, 196). Mineral and bone metabolism disorder has not been completely resolved after kidney transplantation, and risk factors for cardiovascular events continue to exist (197). This may be related to the accumulation of local calcium phosphate salts in renal tissue caused by metabolic disorders (198). This may also be related to metabolic disorders caused by taking immunosuppressants after kidney transplantation (199).

For patients with hepatorenal syndrome leading to AKI, a decrease in serum OPN levels after liver transplantation is positively correlated with kidney function recovery (200); this indicates that serum OPN levels can reflect the extent of kidney injury to some degree. A multitranscriptomic bioinformatics study suggested that OPN is a hub gene after kidney ischemia−reperfusion injury (IRI) (201); this may be related to OPN inhibiting the PI3K/AKT signaling pathway, promoting renal cell death, and exacerbating ischemia−reperfusion injury (202). However, this is not the primary function of OPN; more studies have focused on the role of OPN and immune cells in kidney diseases.

OPN can participate in acute tissue injury by promoting neutrophil chemotaxis (203). Cen et al. (204) reported that treating AKI mice with anti-OPN antibodies not only reduced cell apoptosis and histopathological damage but also decreased NK cell and neutrophil infiltration and decreased IL-6/TNF-α expression. Similar findings were observed in studies on cardiac IRI, where silencing OPN alleviated myocardial I/R injury by inhibiting oxidative stress, myocardial fibrosis, inflammatory responses, and myocardial cell apoptosis (205). These studies indicate that OPN can promote AKI through multiple mechanisms. However, OPN can induce immune cell infiltration and tissue damage not only in AKI but also in chronic kidney disease (CKD), thereby promoting kidney damage.

CKD is typically characterized by proteinuria, loss of kidney function, renal fibrosis, and macrophage infiltration in the kidneys (206). The OPN protein, particularly the OPN-N-terminal protein, is significantly increased in damaged renal tubules and is secreted into the urine via exosomal membranes (207, 208). Exosome-encapsulated OPN can be transferred from damaged renal tubular cells to interstitial fibroblasts through the CD44 receptor, playing a crucial role in renal fibrosis (208). Therefore, detecting OPN levels in urine may offer a noninvasive way to monitor CKD status. Al-Malki (209) reported that the urinary OPN content in DN patients was positively correlated with the presence of podocytes, confirming the feasibility of assessing kidney disease status by measuring urinary OPN levels. Steinbrenner et al. (210) also analyzed urine from CKD patients and reported that high levels of OPN in the urine were associated with worsening kidney function and poor prognosis. Consequently, Owens et al. (211) proposed a CKD progression prediction model based on creatinine, OPN, BUN, and other indicators and found that the model predicted CKD progression with an accuracy of 84.3%. These studies collectively confirm that urinary OPN levels can serve as an effective biomarker for CKD and indicate a correlation between OPN and kidney damage.

In addition, OPN can influence crystal formation in the kidneys through the RGD domain, leading to morphological damage to renal tubular cells. The use of OPN antibodies can reduce the early formation of kidney stones (58). Therefore, OPN is also considered to be significantly associated with urolithiasis (212, 213). This effect may also be achieved through OPN promoting the M1 phenotype of macrophages (214). A large clinical study revealed that the levels of full-length OPN in the urine of patients with urolithiasis were significantly greater than those in healthy controls (215).

The close relationship between the kidneys and the immune system plays a key role in the development of various kidney diseases. As a central molecule, OPN exhibits complex dual mechanisms in different kidney disease contexts. While OPN promotes tumor progression and the formation of an immunosuppressive microenvironment in renal tumors, it may exacerbate inflammatory responses and disease progression in immune-related and other kidney diseases. The source, subtype, cleavage forms, and functional differences of OPN under specific pathological conditions could be focal points for future research.

### 4.2 OPN and kidney transplantation

Kidney transplantation is the best treatment option for end-stage renal disease. Despite the continuous development of antirejection drugs, the incidence of acute rejection remains high, and chronic rejection is a leading cause of graft failure (216, 217). Acute rejection is usually initiated by T cell infiltration and involves inflammation of the interstitium, renal tubules, and microarteries (218, 219). OPN may play a key role in acute rejection because the essence of rejection is a strong immune response (220–223). As a chemokine, OPN can recruit and activate these cells, promoting their infiltration and activation at the site of inflammation (136–138). OPN expressed by tubular epithelial cells can mediate the infiltration of interstitial monocytes, thereby promoting acute kidney transplant rejection (224).

During acute rejection, the expression of CD44 and its ligand OPN in the transplanted kidney is upregulated, indicating increased immune cell infiltration (225). Typically, OPN expression is increased within one week after transplantation, and the use of dexamethasone can inhibit its activation, thereby improving the immune prognosis of the graft (226). When OPN activation is inhibited, the immune prognosis of the graft improves. Additionally, transplanting kidneys from OPN knockout mice into normal mice can significantly reduce graft damage (227). Therefore, OPN may be an important biomarker for predicting rejection or other kidney diseases (13, 19, 228, 229).

OPN has significant diagnostic value for acute rejection (AR). Wang et al. reported that plasma and urine OPN levels were significantly elevated in patients with AR and were positively correlated with Banff grade, indicating that OPN is a reliable biomarker for AR (230). Jin et al. (231) also observed that early in renal acute rejection, blood levels of the kidney injury markers KIM-1 and OPN increased. Xu et al. (232) added HLA-G to blood KIM-1 and OPN markers and reported that the combination of these three markers resulted in a sensitivity of 68.8% and a specificity of 88.9% for predicting AR. These studies confirmed that AR upregulates OPN expression, validating the feasibility of using OPN as a biomarker for acute rejection.

OPN is a predictor of not only AR but also the recovery of renal function after kidney transplantation. The levels of OPN in donor serum and urine and even preservation fluid of the graft may be indicators of patient prognosis (13). A multicenter clinical study revealed that the levels of five proteins, including OPN, in kidney preservation fluid, combined with recipient BMI, could predict short-term renal function after kidney transplantation in a simple, safe, and efficient manner (233). The accuracy of this method was better than that of current prediction algorithms based solely on clinical parameters. In a study on heart transplantation, it was also found that elevated OPN protein levels in organ preservation fluid were positively correlated with severe IRI and the inflammatory response posttransplantation (234). However, there are different conclusions. Mansour et al. (235) analyzed proteins in donor urine and found that a UMOD ratio ≤ 3 had a protective effect on the graft, with a lower risk of DGF; that is, the greater the OPN expression is, the lower the risk of DGF. These two studies measured OPN in preservation fluid and donor urine. The different conclusions might be due to the significant differences in the study samples.

Despite these promising findings, several challenges remain in the clinical application of OPN as a biomarker for AR and other kidney diseases. One significant obstacle is the technical limitations in accurately detecting OPN levels in clinical settings. Current detection methods, such as enzyme-linked immunosorbent assay (ELISA), may struggle with issues such as low sensitivity and specificity, particularly when assessing OPN in complex biological fluids like plasma and urine. To address these challenges, high-throughput screening techniques, including multiplex assays and next-generation biosensors, hold the potential to overcome the limitations of traditional detection methods. However, further validation and standardization of these technologies are necessary before they can be widely implemented in clinical practice.

In addition to playing a role in AR, OPN also has important functions in chronic rejection and the long-term prognosis of kidney transplants. OPN levels in patients after kidney transplantation are positively correlated with serum creatinine levels and negatively correlated with the glomerular filtration rate (236). This finding indicates the potential of OPN as a predictor of long-term prognosis in kidney transplant patients. Chronic rejection, which is usually antibody-mediated, involves various mechanisms, such as APC infiltration and B cell activation (221–223). A single-cell transcriptomics study revealed that during ABMR in the kidney, there is significant infiltration of M1 macrophages in renal tissue while there are almost no M2 macrophages (237). M1 macrophages mediate the infiltration of cytotoxic T cells into the kidneys by secreting various proinflammatory cytokines, causing further damage. The presence of other immunosuppressive cells secreting anti-inflammatory cytokines, such as IL-10, may inhibit cytotoxic T cell activity and infiltration (238).

In addition to macrophages, NK cells may also play an important role in chronic rejection. In studies on chronic rejection, OPN was shown to modulate the immune response and damage renal tubular epithelial cells through NK cells (227). This may be because NK cells activated by OPN upregulate the expression of CD44 and IFN-γ, thereby activating NK cells and exerting downstream effects (239). Additionally, NK cells can secrete various inflammatory mediators, including OPN, which chemotactically attract and activate neutrophils, leading to further kidney damage (240, 241).

The long-term prognosis of a kidney transplant is related not only to chronic rejection but also to chronic fibrosis. The profibrotic effects mediated by OPN result in the formation of scar tissue rather than the effective regeneration of tissue cells (242, 243). Therefore, even long after kidney transplantation, OPN can damage kidney function through this mechanism. OPN expressed by the kidneys or macrophages can be chemotactically attracted by local CCL4 ligands, leading to the infiltration of profibrotic macrophages into the area and promoting their activation and differentiation (244–246). Kidneys with high OPN expression can promote the formation of new lymphatic vessels, providing a pathway for the infiltration of monocytes or other immune cells (247). This infiltration can also promote renal fibrosis. Thus, reducing OPN secretion can slow the progression of fibrosis after transplantation (248). This is likely one of the main reasons that OPN mediates the long-term prognosis of kidney transplants.

Additionally, OPN can serve as a biomarker for graft-versus-host disease (GVHD) after kidney transplantation (249, 250). Graft-derived lymphocytes can exert effects on intestinal epithelial cells by highly expressing OPN, leading to cell death via CD44 on intestinal epithelial cells (106). Studies using ATG to treat GVHD have also shown that OPN levels are significantly elevated during GVHD, suggesting its potential as a biomarker for GVHD (251). When OPN is knocked out, the expression levels of interferon-γ, TNFα, and IL-17 in the small intestine change significantly (252). Therefore, in T cell-mediated acute GVHD, OPN may regulate intestinal cell apoptosis through the Fas-Fas ligand pathway (252). Research by Neo et al. revealed that low OPN expression during GVHD can have anti-inflammatory effects and improve patient prognosis (253). In GVHD, the specific mechanism by which OPN exerts its effects may involve the synergistic action of the T cell receptor/CD3 signaling pathway, which promotes the activation, migration, and infiltration of CD8+ T cells (134). Thus, OPN could be a potential therapeutic target for preventing GVHD.

OPN can also play a role in acute respiratory failure in patients after kidney transplantation. Khamissi et al. (190) found that kidney injury could lead to secondary lung damage and identified kidney-released OPN as a mediator of AKI-ALI through cross-organ, kidney-ligand, and lung-receptor pairing analysis. Increasing evidence shows that extracellular vesicles (exosomes) play a crucial role in cell communication and interorgan interactions (254). The authors suggested that OPN released from renal tubular cells induces lung endothelial cell leakage, inflammation, and respiratory failure. Inhibiting OPN through drugs or gene intervention can prevent AKI-ALI. Another study revealed that allogeneic kidney transplantation can cause distal lung injury in recipients through endoplasmic reticulum stress and necroptosis signaling pathways, with kidney-released OPN being a key factor (255).

However, contrary studies have suggested that OPN is active in MSCs and TSCs, providing support and nourishment to these cells and promoting the proliferation and migration of MSCs (256); this may also be related to the involvement of OPN in the lungs in functions and networks associated with angiogenesis, cell death, and proliferation, thereby promoting acute lung injury after kidney transplantation (257). In OPN-knockout mice, CD4+ T cells were significantly suppressed, and IL-17 expression was markedly downregulated; the authors suggested that OPN can mediate lung inflammation and injury by influencing CD4+ T cells and IL-17 (258). In addition to mediating lung injury, kidney-derived OPN has been shown to promote cardiovascular disease through NLRP3 inflammasome-mediated atherosclerosis (259, 260). Mace et al. (261) studied CKD rats and reported that during CKD, serum OPN levels significantly increase, promoting cardiovascular disease and bone metabolism disorders in rats. Although this study has not yet been extended to organ transplant patients, it may be a potential research direction in the future.

Some diseases caused by abnormal OPN levels, such as atherosclerosis, begin to resolve after kidney transplant function recovery as OPN levels decrease. For example, Renata et al. (262) reported that in chronic kidney disease patients receiving kidney transplants, the levels of OPN in biopsy bone tissue were lower than pretransplant OPN levels. As OPN expression is downregulated, bone-related diseases in kidney transplant patients also improve (263). Liu et al. (264) also reported that after kidney transplantation, the expression of OPN in the arterial wall significantly decreased. This reduction in OPN levels can lead to a decrease in atherosclerosis and cardiovascular events.

Although OPN levels are lower after kidney transplant than before kidney transplant, long-term kidney transplant patients often still suffer from cardiovascular sclerosis-related diseases. This may be related to the activation of the mTOR signaling pathway caused by glucose metabolism disorders induced by steroid medications (265). However, for kidney transplant patients undergoing antirejection treatment with sirolimus, sirolimus can significantly downregulate OPN expression and reverse the adverse cardiovascular effects of glucose metabolism products by inhibiting the mTOR signaling pathway (265).

OPN has significant clinical implications for kidney transplant rejection. By promoting the chemotaxis and activation of immune cells such as T lymphocytes, NK cells, and macrophages, OPN can promote both acute and chronic rejection, affecting graft function and survival. The expression levels of OPN are closely related to the severity of rejection, making OPN in blood and urine a potential biomarker for rejection. Additionally, OPN can be a potential therapeutic target for preventing or treating posttransplant rejection by regulating immune responses. However, there are some discrepancies in the conclusions of different studies regarding the role of OPN. To further apply OPN in clinical settings, it is necessary to elucidate the specific subtypes and cleavage forms of OPN that perform various physiological functions.

Although OPN is activated through enzymatic cleavage, it is also a highly glycosylated and phosphorylated protein. However, in kidney transplantation and AKI, the glycosylation and phosphorylation levels of OPN have not been well studied. The impact of OPN glycosylation and phosphorylation on disease prognosis could be a potential research direction in the future.

## 5 Summary

In the complex immunological environment of kidney transplantation, OPN has garnered widespread attention for its key roles in regulating immune cell activation and influencing inflammatory responses. OPN can be expressed by various cells, such as renal tubular epithelial cells, tumor cells, or macrophages, and exists in five splice variants. The primary domains of the OPN protein, the RGD and SVVYGLR sequences, are usually embedded in the central part of the protein. After cleavage by thrombin or MMPs or binding with heparin, these two important domains are exposed, allowing OPN to bind to receptors such as CD44 and integrins, thereby exerting various physiological functions.

After kidney transplantation, OPN in the kidneys can mediate the activation and infiltration of T lymphocytes, macrophages, and NK cells in renal tissue, thereby regulating acute and chronic rejection and affecting the long-term function of the transplanted kidney. Exosomes can encapsulate OPN, leading to OPN excretion it into the urine, thus allowing noninvasive urine testing. Therefore, OPN can be used as a biomarker for rejection after organ transplantation. Additionally, OPN is a very promising therapeutic target. Therapies targeting OPN, such as the use of specific antibodies and small molecule inhibitors to intervene in OPN-related signaling, offer new strategies to mitigate posttransplant immune responses and promote long-term immune tolerance.

OPN exhibits immunological duality, mediating either an immunosuppressive microenvironment or promoting inflammatory responses depending on the disease context. This may be related to the various splice variants and activation forms of the OPN protein. However, most recent research has not specified splice variants and cleavage forms of OPN. Therefore, future research on OPN should aim to specify the subtypes, cleavage forms, or functional domains of OPN to better elucidate the complex biological roles of OPN.

A deeper understanding of the duality and complexity of OPN in immunology may enable the development of new antirejection methods targeting OPN, thereby improving graft survival rates and patient quality of life. With advances in biotechnology and molecular biology, research on OPN and its related pathways will continue to be an important direction in transplant medicine and immunoregulation studies, leading to innovative progress in the field of kidney transplantation.
